# A new immune signature for survival prediction and immune checkpoint molecules in non-small cell lung cancer

**DOI:** 10.3389/fonc.2023.1095313

**Published:** 2023-01-30

**Authors:** Shuai Han, Dongjie Jiang, Feng Zhang, Kun Li, Kun Jiao, Jingyun Hu, Haihan Song, Qin-Yun Ma, Jian Wang

**Affiliations:** ^1^ Department of Orthopedics, Shanghai Pudong New Area People’s Hospital, Shanghai, China; ^2^ Department of Orthopedic Oncology, Shanghai Changzheng Hospital, Shanghai, China; ^3^ Central Lab, Shanghai Key Laboratory of Pathogenic Fungi Medical Testing, Shanghai Pudong New Area People’s Hospital, Shanghai, China; ^4^ Department of Thoracic Surgery, North Branch of Huashan Hospital, Fudan University, Shanghai, China

**Keywords:** signature, prognosis, immune checkpoint molecules, prediction, non-small cell lung cancer

## Abstract

**Background:**

Immune checkpoint blockade (ICB) therapy has brought remarkable clinical benefits to patients with advanced non-small cell lung carcinoma (NSCLC). However, the prognosis remains largely variable.

**Methods:**

The profiles of immune-related genes for patients with NSCLC were extracted from TCGA database, ImmPort dataset, and IMGT/GENE-DB database. Coexpression modules were constructed using WGCNA and 4 modules were identified. The hub genes of the module with the highest correlations with tumor samples were identified. Then integrative bioinformatics analyses were performed to unveil the hub genes participating in tumor progression and cancer-associated immunology of NSCLC. Cox regression and Lasso regression analyses were conducted to screen prognostic signature and to develop a risk model.

**Results:**

Functional analysis showed that immune-related hub genes were involved in the migration, activation, response, and cytokine-cytokine receptor interaction of immune cells. Most of the hub genes had a high frequency of gene amplifications. MASP1 and SEMA5A presented the highest mutation rate. The ratio of M2 macrophages and naïve B cells revealed a strong negative association while the ratio of CD8 T cells and activated CD4 memory T cells showed a strong positive association. Resting mast cells predicted superior overall survival. Interactions including protein–protein, lncRNA and transcription factor interactions were analyzed and 9 genes were selected by LASSO regression analysis to construct and verify a prognostic signature. Unsupervised hub genes clustering resulted in 2 distinct NSCLC subgroups. The TIDE score and the drug sensitivity of gemcitabine, cisplatin, docetaxel, erlotinib and paclitaxel were significantly different between the 2 immune-related hub gene subgroups.

**Conclusions:**

These findings suggested that our immune-related genes can provide clinical guidance for the diagnosis and prognosis of different immunophenotypes and facilitate the management of immunotherapy in NSCLC.

## 1 Introduction

Lung cancer is the most common malignant tumor in China and worldwide, which has the highest mortality rate for both men and women and is responsible for 23% of cancer-associated related deaths (1). Non-small cell lung cancer (NSCLC) accounts for more than 85% of lung cancer cases and can be further divided into three subtypes: lung adenocarcinomas (LUAD), squamous cell carcinomas, and large cell carcinomas (2).With the decreasing of smoking rates, LUAD has become the most prevalent histological subtype. LUAD is characterized by high metastasis rate and notable invasiveness. Despite new developments in surgery, chemotherapy, radiotherapy, and molecularly targeted therapy, the prognosis for LUAD patients remains unsatisfactory, with a 5-year survival rate of around 18% (3).

With rapid advancement of precision medicine, the clinical benefits of checkpoint blockade therapy have rekindled the hope for better outcome of LUAD immunotherapy. Immune checkpoint inhibitors target tumor-specific antigens which are utilized by cancer cells to evade tumor-reactive immune cells. To date, immune checkpoint molecules mainly include programmed cell death protein 1 (PD-1), mucin domain-containing 3 (TIM3), lymphocyte-activation gene 3 (LAG3), and cytotoxic T-lymphocyte antigen-4 (CTLA-4). Antibodies blocking PD1/PDL1 have been approved for clinical use and have received impressive clinical responses in some patients with LUAD (4–11). Unfortunately, only around 20% of NSCLC patients benefit from anti-PD-1/PD-L1 therapy. It has been speculated that the heterogeneity of LUAD and the tumor microenvironment may contribute to the diverse antitumor immune responses (12).Thus, regarding their prognostic potential in LUAD, the molecular events of tumor cell immunocyte interactions in LUAD microenvironments need to be further summarized and the expression level of immune related genes (IRGs) in LUAD needs to be comprehensively explored (13).

Although there have been some research findings regarding IRGs and LUAD prognosis, they were focused on single biomarkers and a prognostic model based on IRGs that can systematically assess the prognosis of LUAD patients is not available (1, 2, 6, 13). Therefore, there is an urgent need to construct a robust and simple IRG prognostic signature model.

In this study, we combined all known IRGs from multiple immunology databases and then performed weighted gene co-expression network analysis (WGCNA) to identify hub IRGs in TCGA-LUAD cases. After that, we evaluated the mutation rate of the hub IRGs and tried pathway and GO enrichment. Then, we evaluated immune cell infiltration by using CIBERSORT and merged the results with hub IRGs for correlation analysis. Next, we selected IRGs associated with the survival outcome of LUAD patients and constructed a gene prognostic model based on Lasso Cox regression. Finally, we clustered hub IRGs in an unsupervised fashion and compared the differences of immune cell infiltration as well as the sensitivity to anticancer drugs in different groups. These could be ultimately used to assist clinicians in prognostic evaluation and therapeutic selection of LUAD patients and to provide further insights into the molecular mechanism of immune-related genes in tumor immune evasion.

## 2 Methods

### 2.1 Downloading of data source and clinical information

**Figure 1:**
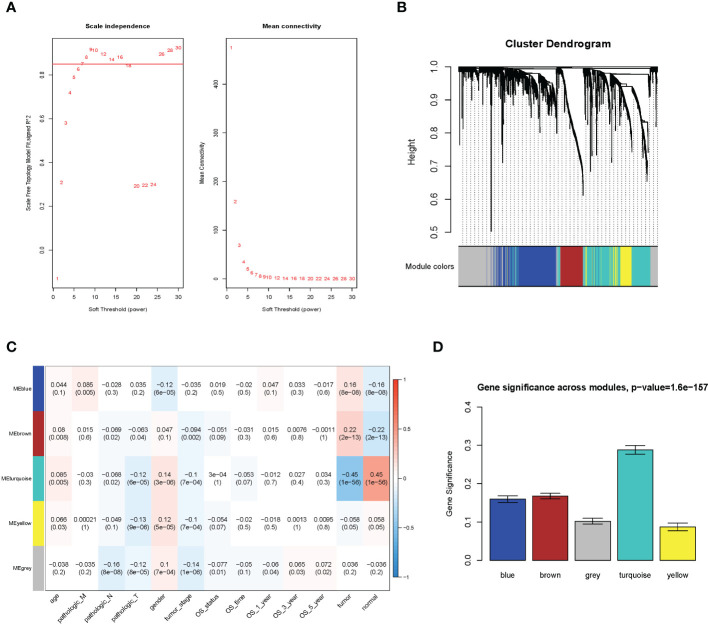
WGCNA analysis result. **(A)** Soft threshold; **(B)** cluster analysis; **(C)** correlation analysis between different modules and sample traits; **(D)** average value of gene significance (GS) across module.

The overall analysis scheme is illustrated in **Figure 1**. The mRNA expression, somatic single-nucleotide mutation, and clinical data of TCGA-LUAD and TCGA-LUSC were downloaded from the UCSC Xena database. Immune-related genes were downloaded and merged from the InnateDB, Immport, and IMGT/GENE-DB databases. Then, we combined the gene expressions and acquired the immune-related gene expression matrix.

### 2.2 Weighted gene co-expression network analysis and identification of key modules and hub genes

The WGCNA methodology analysis was performed according to Langfelder’s instructions. We used R package WGCNA 1.69 to identify the crucial immune-related gene modules. The expression matrix was confined to only immune-related genes. The soft threshold was calculated, and the screening threshold was set as R^2^ >0.85 (power = 7). Then, the one-step function was used for network construction and detection of consensus modules. Similar modules were clustered and merged in accordance with the threshold of height less than 0.25. Finally, we obtained four modules and associated molecular features with clinical information for predicting outcomes for LUAD patients. The turquoise molecular structure had the strongest association with the prognosis and was selected for further analysis.

### 2.3 Functional analysis and mutation analysis of hub genes

We selected the hub gene from the turquoise module based on the values of GS and MM (GS >0.3, MM >0.5). Functional enrichment analysis was performed using R package cluster Profiler, and the threshold sets were p-value <0.05 and q-value <0.2. We also conducted mutational analysis of hub genes, and genes with a mutation rate of more than 5% were exhibited.

### 2.4 Tumor immune infiltration analysis

We used the R package CIBERSORT to evaluate 22 immune cell types in each sample of the LUAD cohort. Samples with p < 0.05 were considered eligible and used for subsequent studies. Kaplan–Meier survival analysis was applied first to explore the prognostic value of tumor-infiltrating immune cells. Then, Pearson’s correlation test was performed to evaluate the correlation between tumor-infiltrating immune cells and the correlation between tumor-infiltrating immune cells and hub genes.

### 2.5 Construction of the lncRNA/mRNA/TF co-expression network based on hub genes

In brief, interaction network data were downloaded from RAID and TRRUST databases. LncRNAs, mRNAs, and TFs, which are associated with hub genes, were extracted and introduced into Cytoscape 3.71 to generate the interaction network.

### 2.6 Construction and validation of an immune prognostic model for LUAD

The prognostic model was developed in the following steps. First, univariate Cox analysis was used to determine the connection between hub genes and prognosis. Genes with p-value <0.05 were selected. Then, Lasso regression was performed to remove highly correlated genes and build survival models. Patients were divided into high-risk and low-risk groups, and Kaplan–Meier survival curves were plotted. Next, the area under the receiver operating characteristic curves (AUC) at different cutoff values of overall survival time was calculated to evaluate model discrimination. Finally, independent GEO LUAD dataset GSE30219 was used to further validate the prognostic value of our model.

### 2.7 LUAD molecular subtypes based on unsupervised hierarchical clustering

We also divided patients into two groups through unsupervised clustering analysis of hub genes. We first compared the survival curves between the two groups. Then, we used a heatmap to show the distribution of tumor immune cell infiltration between the two groups. Finally, we evaluated the efficacy of immunotherapy and drug sensitivity between the two subgroups by using Tumor Immune Dysfunction and Exclusion (TIDE) web application (http://tide.dfci.harvard.edu) and the R package “pRRophetic,” respectively.

## 3 Results

### 3.1 Data downloading and integration

Gene expression, phenotype, and clinical data of LUAD and LUSC were downloaded from the UCSC Xena database. After removing the missing samples of clinical data, we collected a total of 1,114 samples, including 1,006 cancer samples and 108 pericarcinomatous samples. A total of 3,511 IRGs were screened out from the InnateDB, Immport, and IMGT/GENE-DB databases. Combined with the expression data, the expression matrix of 2,531 IRGs was finally obtained.

### 3.2 WGCNA

We utilized the WGCNA to construct the link among the 2,351 immune-related genes from 1,114 NSCLC samples. A total of five modules were obtained through a one-step network construction method, where power = 7. Then, we performed a correlation analysis between different modules and sample traits. The distributions of the modules’ average gene significance related to OS were identified, among which the turquoise module (including 709 genes) was found to have the strongest association with the sample feature. This module was selected for further analysis (**Figure 1**).

### 3.3 Hub gene selection

The GS value and module membership (MM) value of genes in the turquoise module were calculated. There were 280 hub genes screened by the threshold of GS >0.3 and MM >0.5. (**Supplementary Figure 1**).

### 3.4 Functional enrichment analysis (GO/KEGG)

Gene ontology (GO) enrichment analysis was divided into three categories: Biological Process (BP), Cellular Component (CC), and Molecular Function (MF). The BP enrichment pathway was mainly of regulation of inflammatory response, positive regulation of response to external stimulus, and leukocyte migration. The CC enrichment pathway was mainly of the external side of the plasma membrane, tertiary granule, and secretory granule membrane. The MF enrichment pathway was mainly of cytokine binding, G protein-coupled peptide receptor activity, and cytokine receptor activity. The KEGG enrichment pathway was mainly of cytokine–cytokine receptor interaction, *Staphylococcus aureus* infection, and phagosome. These pathways suggested that the related genes mainly functioned by regulating immune reaction (**Supplementary Figure 2**).

### 3.5 The characteristic of hub genes by the whole genome

Samples of lung adenocarcinoma (TCGA, Firehose Legacy) and lung squamous cell carcinoma (TCGA, Firehose Legacy) were selected from the cBioPortal database. Mutations were detected in 280 genes. A total of 54 genes had a mutation rate of over 5%. The mutation rate of MASP1 was the highest (22%) according to the gene mutation map, followed by SEMA5A (18%). All the mutational genes in the map were associated with NSCLC (**Supplementary Figure 3**).

### 3.6 Analysis of immune cell infiltration

The ratios of 22 infiltrated immune cells in cancer samples were calculated. According to the proportion level of infiltrated immune cells in cancer samples, we calculated the Pearson correlation coefficient between the different infiltrated immune cells and drew the heatmap. The results indicated that macrophages M2 and naive B cells showed a significantly negative correlation, whereas CD8+ T cells and activated CD4+ memory T cells, naive B cells, and plasma cells displayed a significantly positive correlation (**Supplementary Figure 4**).

### 3.7 Survival analysis of infiltrated immune cells

**Figure 2:**
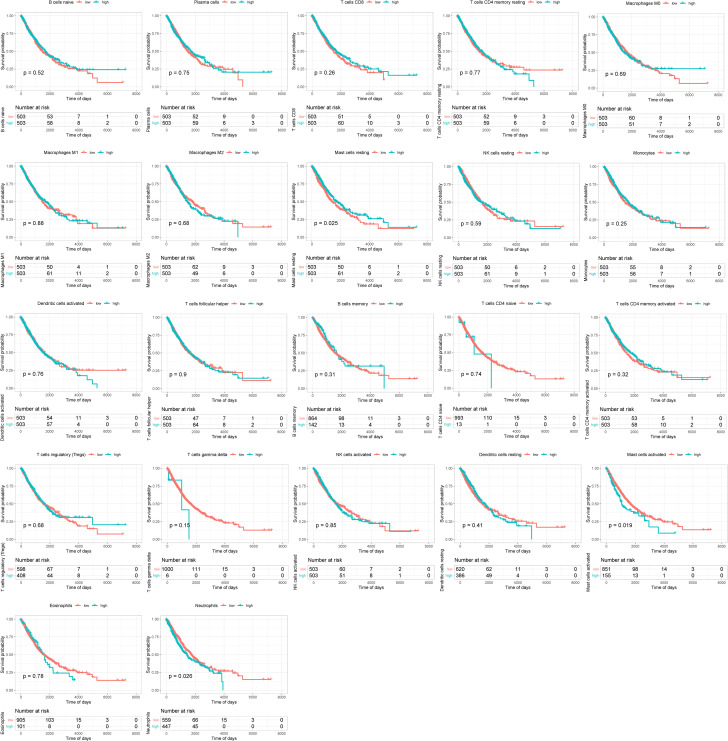
Survival analysis of infiltrated immune cells between high- and low-infiltration groups.

Firstly, we divided the infiltrated immune cells into high- and low-ratio groups based on the median infiltrating level of immune cells. Then, we calculated and drew Kaplan–Meier (KM) survival curves between the two groups based on the survival data. The results revealed that the high-infiltration group of resting mast cells displayed remarkably better overall survival (OS) than those with low infiltration and the high-infiltration group of activated mast cells and neutrophil displayed remarkably worse OS than those with low infiltration (**Figure 2**).

### 3.8 Correlation analysis between infiltrated immune cells and hub genes

**Figure 3:**
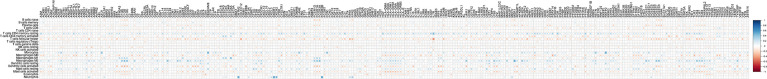
Correlation analysis between infiltrated immune cells and hub genes.

The Pearson correlation coefficient was calculated between hub genes and infiltrated immune cells. The results suggested that almost all the genes were positively associated with resting CD4+ memory T cells and negatively correlated with T follicular helper cells and activated mast cells (**Figure 3**).

### 3.9 Univariate Cox regression analysis

**Figure 4:**
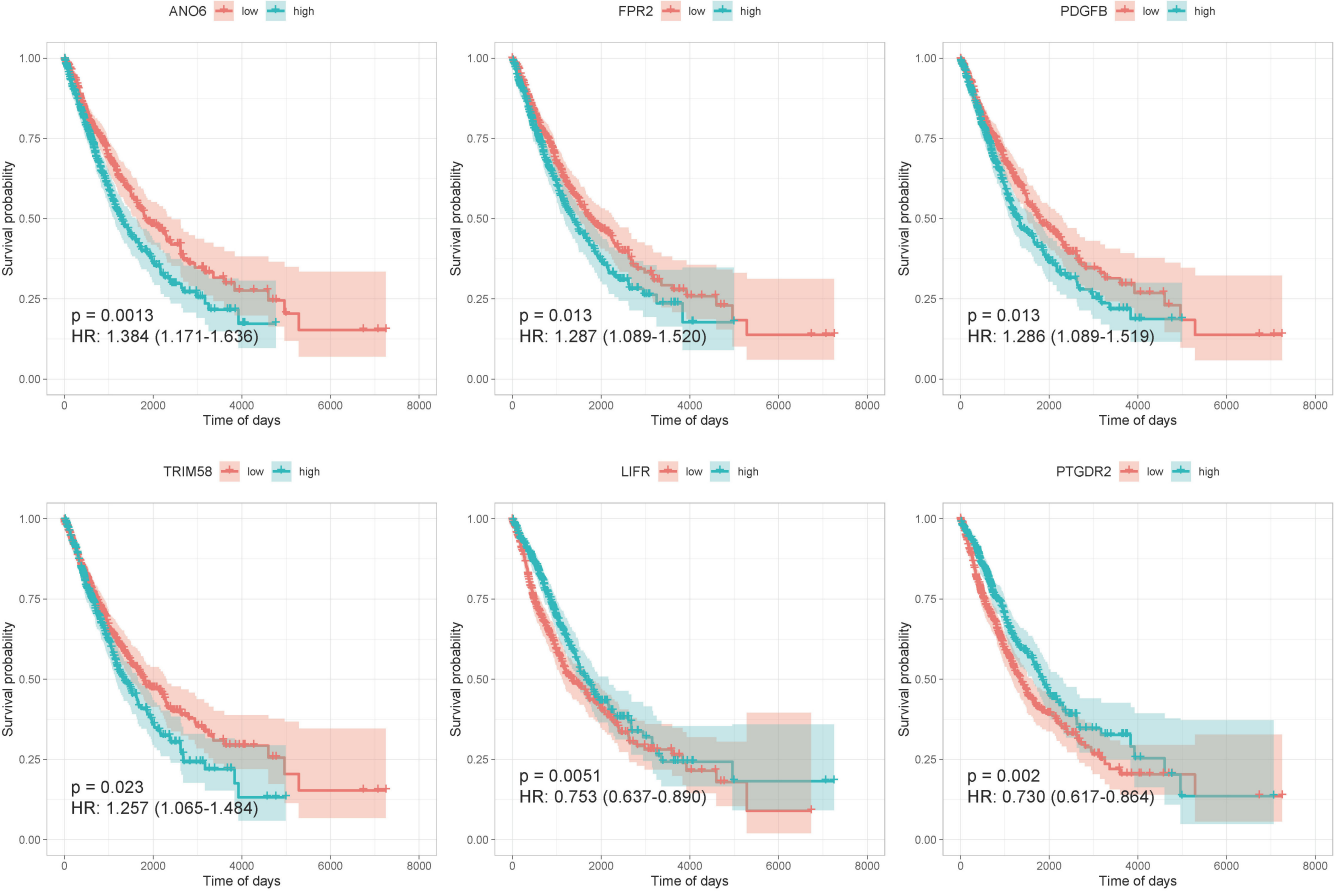
The KM survival curves of six prognosis-related genes.

We constructed a regulatory network based on 181 TFs, 144 lncRNAs, and 424 mRNAs, which were interacting with hub genes from different databases (**Supplementary Figure 5**). After the analysis of univariate Cox regression, a total of 15 prognosis-related genes of NSCLC were selected from an expression matrix of 1,006 cancer samples, such as ANO6, FPR2, PDGFB, TRIM58, CD300E, CXCL3, HLA-DMA, CTSM, ANOS1, NR3C2, BMP5, TLR7, FCGRT, LIFR, and PTGDR2. The KM survival curves were generated in six of them. The curves revealed that the high expression group of ANO6, FPR2, PDGFB, and TRIM58 displayed remarkably worse OS than those with a low expression. The curves also revealed that the low expression group of LIFR and PTGDR2 displayed remarkably worse OS than those with a high expression (**Figure 4**).

### 3.10 Validation of gene prognostic signature

We performed Lasso-penalized Cox regression analysis with cross-validation to pick out nine genes from the 15 candidates. Furthermore, among the nine genes, TRIM58, PDGFB, FPR2, and ANO6 were prognostic risk factors (HR >1), whereas TLR7, PTGDR2, NR3C2, LIFR, and ANOS1 were prognostic protective factors (HR <1). To evaluate the nine-gene prognostic signature, we calculated the risk score for each sample in TCGA according to the expression levels of nine genes weighed by their relative coefficient using the following formula: risk score = PTGDR2*(-0.140) + ANOS1*(-0.115) + LIFR*(-0.091) + TLR7*(-0.052) + FPR2*(-0.026) + NR3C2*(0.020) + PDGFB* (0.090) + TRIM58*(0.127) + ANO6*(0.176). The risk scoring section of each sample was (-0.762–1.910), and high-/low-risk groups were divided with the median of risk score as the cutoff (**Supplementary Figure 6**).

**Figure 5:**
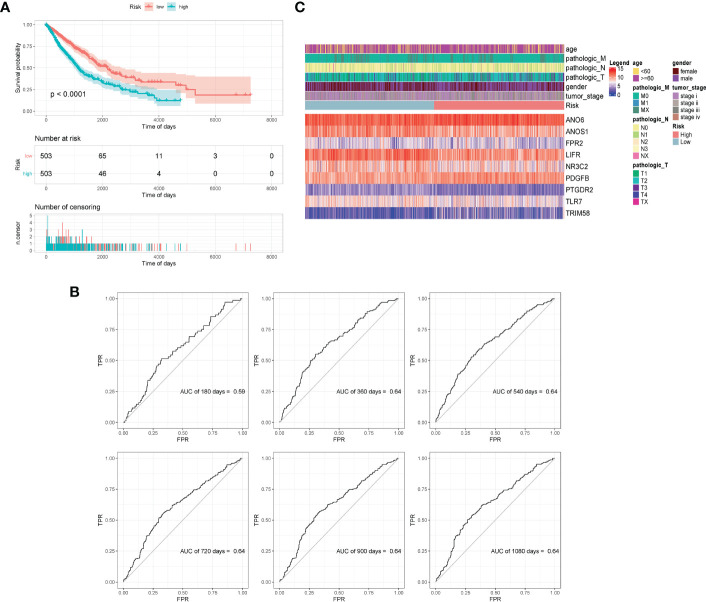
Validation of model effectiveness. **(A)** KM curves of high- and low-risk scores. **(B)** The AUC value of 180d, 360d, 540d, 720d, 900d, and 1080d by ROC curves. **(C)** Heatmap of gene expression in model.

All samples were separated into high- or low- risk groups according to the median of risk score. K–M curves showed that patients in high-risk group had significantly worse prognosis than those in the low-risk group (log-rank, p < 0.0001), which suggested that the model had favorable efficiency. ROC curves were also applied for the prognosis of samples depending on risk scores. The AUC values of 360d, 540d, 720d, 900d, and 1080d were all above 0.6, whereas the value of 180d was 0.59 (**Figure 5**).

**Figure 6:**
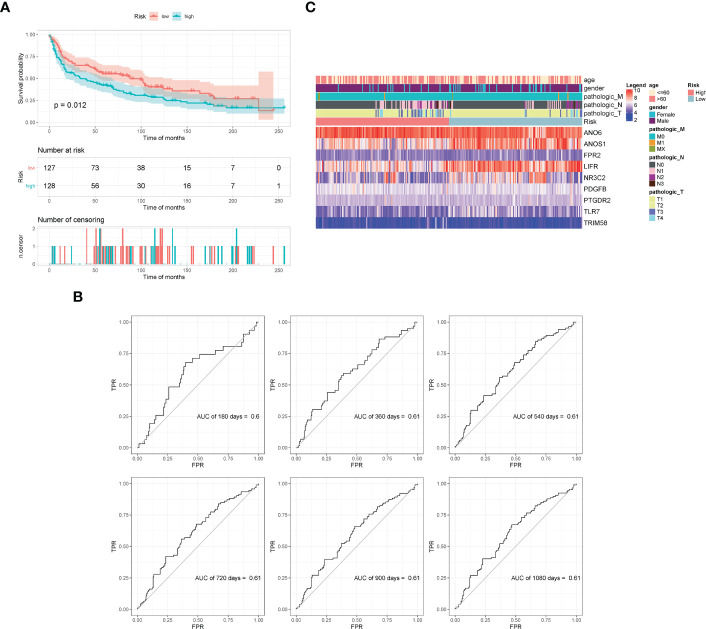
Validation of model effectiveness by dataset. **(A)** KM curves of high- and low-risk scores. **(B)** The AUC values of 180d, 360d, 540d, 720d, 900d, and 1080d by ROC curves. **(C)** Heatmap of gene expression in the model.

The GSE30219 dataset was selected as the validation set to further verify the prognostic predictive value of the nine-gene signature. Survival analysis of the validation dataset also showed a significant difference in OS between the high- and low-groups (p = 0.012). The AUC values of 180d, 360d, 540d, 720d, 900d, and 1080d were all above 0.6 according to ROC curves (**Figure 6**). The results demonstrated that the nine-gene signature was reliable and effective for prognostic prediction in NSCLC.

### 3.11 Classification by unsupervised clustering analysis

We employed an unsupervised clustering algorithm to classify the 1,006 samples of NSCLC patients. They were classified into two clusters, cluster1 with 474 samples and cluster2 with 532 samples. The heatmap showed that the expression of hub genes was different in the two subtypes. Survival analysis showed significant differences between the two clusters (p = 0.048) (**Supplementary Figure 7**).

### 3.12 Immune infiltration landscape and difference in proportion of infiltrated immune cells

We investigated possible clinical factors related to the two clusters, including age, gender, TNM stage, tumor stage, risk score, and immune cell abundance. There were no statistical differences between the two clusters in terms of age, gender, TNM stage, and tumor stage; however, the risk score in cluster1 was higher than that in cluster2.

**Figure 7:**
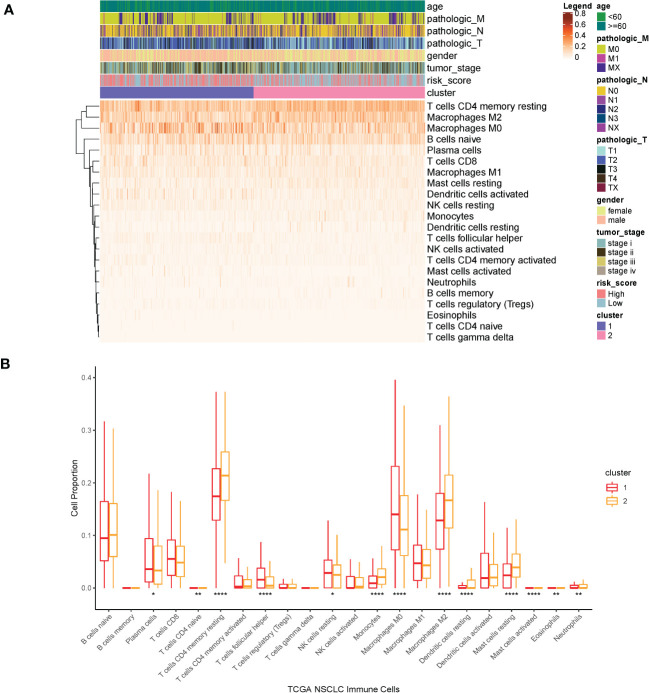
**(A)** Heatmap of clinical factors and infiltrated immune cells in the two clusters. **(B)** Proportion of immune cells in the two clusters. ****p<0.0001, **p<0.01, *p<0.05.

Then, we estimated the proportion of immune cells in the two clusters and found that the tumor infiltration of resting memory T cells, naïve B cells, macrophages M0, and macrophages M2 were highly expressed in all samples. The proportion of macrophages M0 was extremely higher in cluster1 than that in cluster2, whereas the proportion of resting memory T cells and macrophages M2 was extremely lower in cluster1 than that in cluster2 (Kruskal–Wallis, p < 0.0001) (**Figure 7**).

### 3.13 Effect of immunotherapy in different subtypes

An analysis of expression levels for 44 immune checkpoints between the two clusters were performed (**Supplementary Figure 8**). The expressions of almost all the immune checkpoints were significantly different in the two clusters. TIDE scores of the two clusters were calculated by the TIDE online analysis tool. The statistical results showed that the TIDE scores of most samples were lower than 0, which indicated that immunotherapy might be effective for most samples. Furthermore, the TIDE score for samples in cluster1 was extremely lower than that for samples in cluster2 (t-test, p < 0.0001) (**Supplementary Figure 9**).

### 3.14 Chemosensitivity in different subtypes

**Figure 8:**
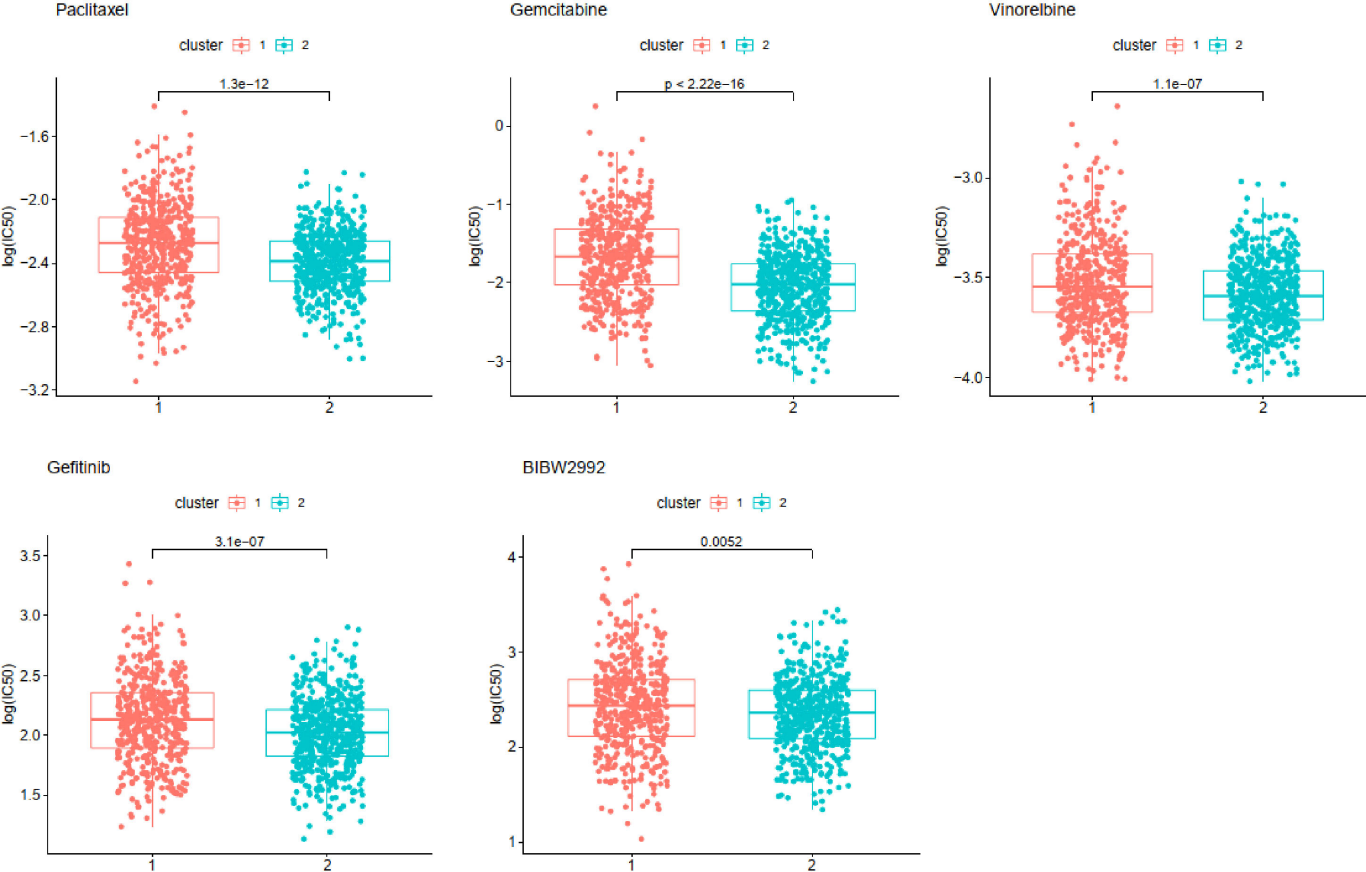
Drugs sensitive in cluster1.

**Figure 9:**
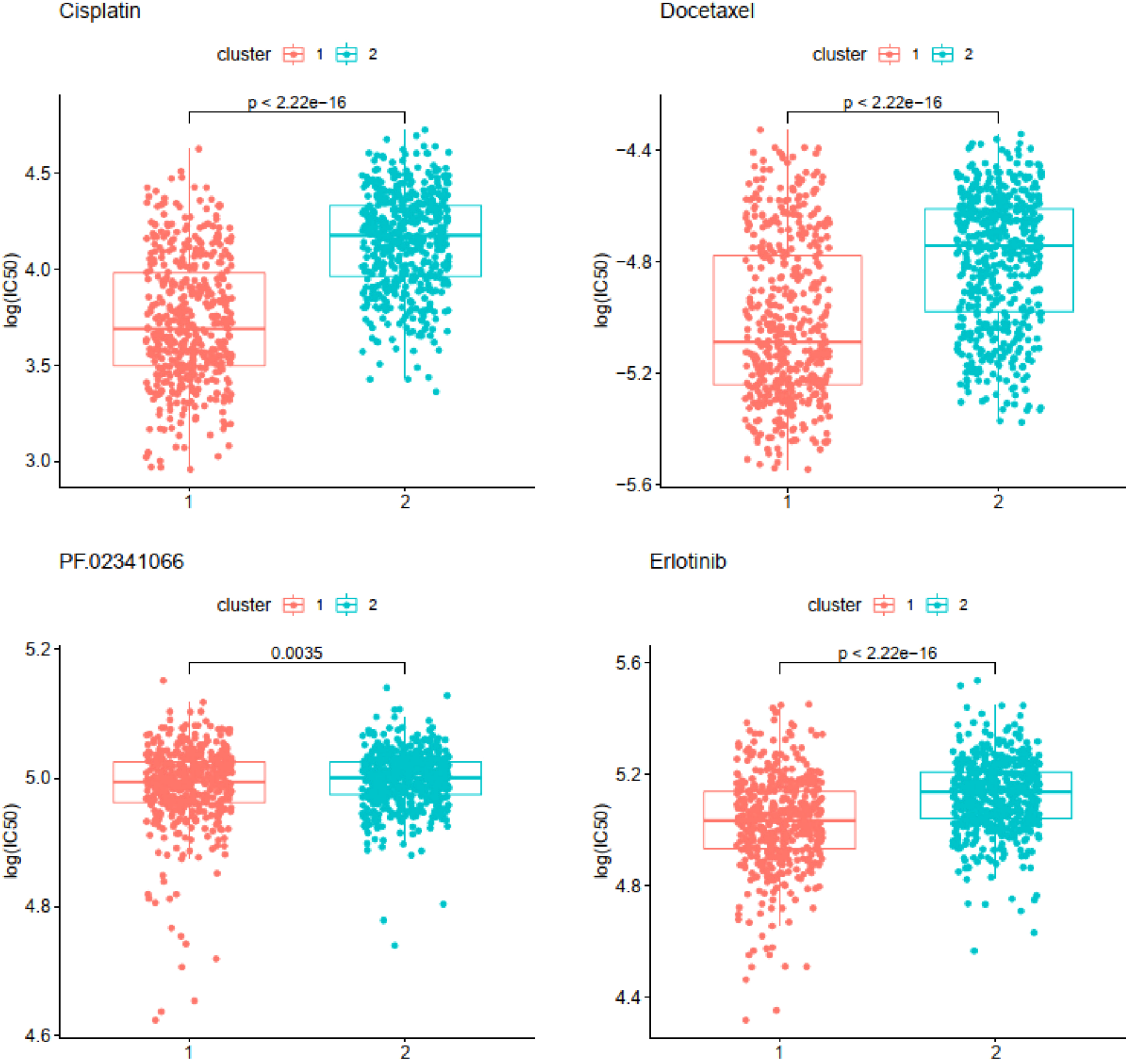
Drugs sensitive in cluster2.

Chemosensitivity prediction was further investigated in the two clusters. The t-test analysis indicated that there was a significant difference between the two clusters in sensitivity. Patients in cluster1 were more sensitive to paclitaxel, gemcitabine, vinorelbine, gefitinib, and afatinib (BIBW2992) (**Figure 8**). However, patients in cluster2 were more sensitive to cisplatin, docetaxel, erlotinib, and crizotinib (PF-02341066) (**Figure 9**). The results could provide a convincing basis for the use of related drugs in treating patients with different clusters.

## 4 Discussion

Lung cancer is the leading cause of cancer-related deaths in the world, with an average 5-year survival rate of 21% (1). Lung cancer initiation and progression depend not only on the evolving genomics and molecular properties of cancer cells but also on their interaction with the tumor environment, specifically with the immune system (4). The immune system is now recognized as having the potential to destroy cancer cells and inhibit tumor growth through the activation of innate and adaptive immune responses; however, the immune system may also promote tumor progression (5).

Based on the statistical analysis of the whole-genome characteristics, we found a total of 54 mutated IRGs with mutation rate >5% and most of them were amplified in the genome. It is confirmed that tumor mutational burden is associated with improved survival in patients receiving immune checkpoint inhibitors across a wide variety of cancer types. The most frequently mutated IRGs is MASP1 (22%), followed by SEMA5A (18%), which has never been reported previously in NSCLC. MASP1 is an abundant component of the lectin pathway of complement (14, 15). The complement pathway plays an essential role in innate and adaptive immune responses. The mutation in MASP1 may cause cancer because of immunological abnormality. Semaphorin 5A, a member of the semaphorin family, plays an important role in axonal guidance. The downregulation of SEMA5A in lung adenocarcinoma tissues was associated with a poor overall survival. A suppressive role for SEMA5A in lung adenocarcinoma involves the inhibition of the proliferation and migration of lung transformed cells (16). Our findings have identified certain mutational characteristics of IRGs in NSCLC, offering new perspectives in the etiology and treatment of NSCLC.

IRGs have been used to predict the prognosis of NSCLC patients in previous research (6, 7, 17). In the present study, nine IRGs associated with cancer prognosis were screened out from TCGA data. Among the nine genes, PDGFB, FPR2, ANO6, and TRIM58 were prognostic risk factors, whereas PTGDR2, ANOS1, LIFR, TLR7, and NR3C2 were prognostic protective factors. PDGFB encodes a member of platelet-derived growth factors, playing a role in a wide range of developmental processes. An investigation on 442 patients with LUAD indicated that a high expression of PDGFB and presentation of mesenchymal-like tumors were significantly associated with poor prognosis for both OS and disease-free survival (18). FPR2 (formyl peptide receptor 2), as a G-protein-coupled receptor, was involved in a broad spectrum of pathophysiologic processes. It was found that a high expression of FPR2 was associated with a lower OS in LUAD patients (8). ANO6 is a member of the TMEM16 family, which was initially discovered as Ca^2+^-activated Cl^-^ion channels (19). The TMEM16 family was found to be overexpressed in cancer cells associated with poor prognosis and cancer development. ANO6 was also associated with metastatic capability of mammary cancers in mice and was related to poor prognosis of patients with breast cancer (20). It has been demonstrated that ANO6 is an essential component of the immune defense by macrophages (21). However, the role of ANO6 in lung cancer has not been illustrated. TRIM58 is a prognostic indicator for LUAD and LUSC. KRAS-driven LUAD samples with a higher expression level of TRIM58 were found to have a relatively high expression level of immune checkpoints genes, including PD-1, PD-L1, and CTLA-4 (22). LUSC patients with high methylation levels of TRIM58 had a longer survival time (23). On the contrary, in our study, TRIM58 was considered as a risk factor for the prognosis of NSCLC patients. In addition, TRIM58 was positively correlated with abundance of M2 macrophages and resting mast cells and negatively correlated with follicular helper T-cell abundance in KRAS-driven LUAD (22). Thus, the role of TRIM58 needs to be further identified.

PGD2/PTGDR2 signaling was found to inhibit tumorigenesis, tumor growth, and metastasis in gastric cancer (24). However, PTGDR2 was rarely reported in lung cancer. CRTh2, encoded by PTGDR2, is preferentially expressed in CD4+ effector T helper 2 (Th2) cells. T-cell activation could reduce the expression of CRTh2 at the level of both transcription and protein expression (9). LIFR, the receptor of the leukemia inhibitory factor, was reported as a prognostic protective factor in LUAD patients (25). A low LIFR expression was associated with shorter survival in pancreatic cancer and NSCLC patients with mutated KRAS (10). TLR7 is expressed on endosomes in immune cells including plasmacytoid and conventional dendritic cells, macrophages, B lymphocytes, and NK cells (26). Stimulation of these immune cells with TLR7 ligands induces their maturation and activation, leading to antitumor therapeutic efficacy in colon, renal, and breast carcinomas. By contrast, several studies have shown that TLR7 is highly expressed in lung cancer cells, leading to increased tumor cell survival, chemoresistance, and poor clinical outcomes (27–29). A genome-wide lethality screening in NSCLC reported that NR3C2 might be a potential tumor-suppressing gene (30). Other researchers also found that NR3C2 was downregulated in metastasis samples and the OS rate in patients with a high expression of NR3C2 was higher than that in patients with a low expression of them in LUAD (31–34).

Infiltrated immune cells in the tumor microenvironment of lung cancer play a key role in tumor progression and have been widely studied in recent years. In our research, we found that the infiltration degrees of mast cells and neutrophils were associated with prognosis of NSCLC. Mast cells are well known for their roles in allergic disorders (35). However, the consequences of their presence in the tumor microenvironment still remain unclear as it is associated with a good or poor prognosis based on the type and anatomical site of the tumor (36). Mast cells through releasing IL-1, IL-4, IL-6, and TNF-α can actively participate in the elimination of tumor cells and rejection of tumors (37). Conversely, mediators released by mast cells such as FGF-2, NGF, PDGF, VEGF, IL-8, and IL-10 can promote the expansion of tumor cells (38). Mast cell infiltration has been implicated in metastasis and angiogenesis in several human malignancies (38). Our results indicated that a high level of resting mast cell infiltration was associated with better prognosis, whereas a high level of activated mast cells was significantly related to worse OS. Neutrophils have been implicated in all stages of the oncogenic process. However, the effect of neutrophil maturity on their antitumor or protumor properties remains understudied. A meta-analysis of nearly 4,000 patients has found high levels of intra-tumoral neutrophils to be associated with unfavorable survival outcomes (39, 40). In accordance with these studies, a high proportion of neutrophils was significantly related to worse OS in our research.

The CD4+ memory T cells were constitutively presented in the microenvironment of lung cancer, which could be mobilized by IL-12 to proliferate and kill tumor cells in the xenograft (41). T follicular helper cells were likely to be involved in the antitumor immunity and were associated with better clinical outcomes in NSCLC (42). For adenocarcinoma patients, memory B‐cell and resting CD4+ T-cell fractions were associated with better OS, whereas the neutrophil, follicular helper cell, M0 macrophage, and M2 macrophage fractions were associated with a shorter OS. For squamous cell carcinoma patients, a higher percentage of regulatory T cells and naïve CD4+ T cells was associated with a marginally poorer overall OS (43). In our research, almost all the hub genes were positively correlated with resting CD4+ memory T cells.

In the present study, we identified a novel and independent classification based on the IRG expression profiles. According to research, the patients in cluster2 had a better OS. Interestingly, we found that the proportion of infiltrated immune cells was remarkably different in the two subtypes, especially in resting memory T cells, macrophages M0, and macrophages M2. However, these immune cells mentioned above presented no difference in OS.

The therapies for NSCLC, including chemotherapies, targeted therapies, and immunotherapy, have undergone great advancements over the past two decades. Cytotoxic therapies have demonstrated a remarkable effect on early-stage NSCLC, whereas adjuvant cytotoxic therapy with a cisplatin-based doublet is associated with improved survival in patients with resected advanced NSCLC (44). The standard therapy for patients with unresectable advanced NSCLC is the combination of cytotoxic therapy and thoracic radiation (45). Molecularly targeted therapies prove to have a good prognosis in non-squamous NSCLC patients with EGFR, ALK, ROS1, BRAF, and NTRK mutations (45–50). However, activating mutations are rare in LUSC and targeted therapies for LUSC patients remain less effective (51). Fortunately, several studies have demonstrated that monotherapies with antibodies against PD-1 or PD-L1 can significantly improve OS for LUAD and LUSC patients (3). Although both PD-1 and TMB may be used to select patients for immunotherapy, most patients will not fit the ideal profile based on these two biomarkers (52). In our study, the two clusters classified by a new method presented different sensitivities to chemotherapy and immunotherapy. The samples of cluster1 were more sensitive to gemcitabine and paclitaxel, whereas the samples of cluster2 were more sensitive to cisplatin, erlotinib, and docetaxel. All the samples might have a high likelihood of responding to immunotherapy. Compared with cluster1, however, the samples of cluster2 seemed to be more sensitive to immunotherapy. The molecular differences between the identified subtypes may facilitate the development of more appropriate therapeutic approaches.

There are still some limitations to the research. Because of retrospective data gained from public databases, the model needs to be further validated by a larger number of clinical samples. Additionally, bias of expression exists in IRGs, which has been caused by heterogeneity in NSCLC.

## 5 Conclusion

The IRGs and the related immune cells may be used to guide prognosis prediction and clinical decisions for NSCLC patients. These findings may be considered as therapeutic targets as well as possible playmakers in the antitumor immune response to newer targeted cancer drugs.

## Data availability statement

The original contributions presented in the study are included in the article/**Supplementary Material**. Further inquiries can be directed to the corresponding authors.

## Author contributions

SH, DJ, FZ, KL, KJ, JH, HS, Q-YM, and JW designed the study. SH, DJ, FZ, KL, KJ, and JH analyzed the data; SH, DJ, FZ, HS, Q-YM, and JW wrote the manuscript. All authors contributed to the article and approved the submitted version.

## Supplementary material

Supplementary Figure 1 Scatter plot of correlation between GS and MM gene in turquoise module.

Supplementary Figure 2 Bubble chart of enrichment analysis, including Biological Process **(A)**, Cellular Component **(B)**, Molecular Function **(C)** and KEGG enrichment pathway **(D)**.

Supplementary Figure 3 Genes of mutation rate > 5%.

Supplementary Figure 4 Correlation analysis of infiltrated immune cells.

Supplementary Figure 5 Construction of multi-factor regulatory network.

Supplementary Figure 6
**(A)** Hazard ratio of 15 genes; **(B)** cross-validation of Lasso-penalized Cox regression analysis; **(C)** 9 genes for prognostic related risk score model.

Supplementary Figure 7 Unsupervised clustering classification based on hub genes. **(A)** Inflection point figure by elbow algorithm; **(B)** Heatmap of gene expression in the two subtypes; **(C)** KM curves between the two clusters.

Supplementary Figure 8 Expression levels of 44 Immune Checkpoints between the two clusters.

Supplementary Figure 9 TIDE scores of samples in the two clusters.
