# Supplementary figures and images for: A new immune signature for survival prediction and immune checkpoint molecules in non-small cell lung cancer
Source: Front Oncol. 2023 Jan 30;13:1095313. doi: 10.3389/fonc.2023.1095313 (PMC9924230; doi:10.3389/fonc.2023.1095313)

**Module membership vs. gene significance**  
**cor=0.3, p=3.3e-16**

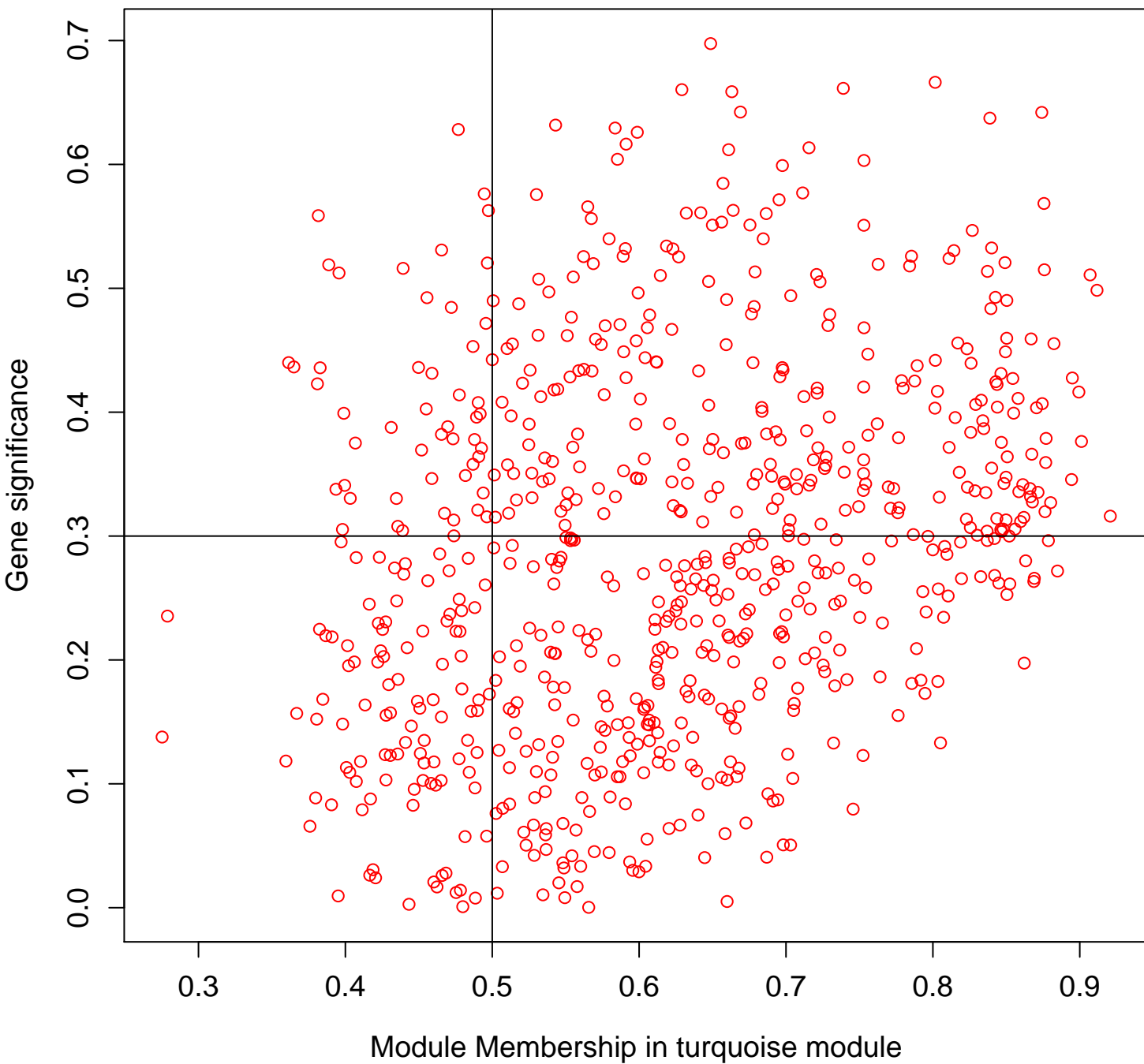

Supplement: Supplementary Figure 1 — Scatter plot of correlation between GS and MM gene in turquoise module. [file DataSheet_1.zip › Supplementary Figures/Supplementary Figure 1.PDF]

A

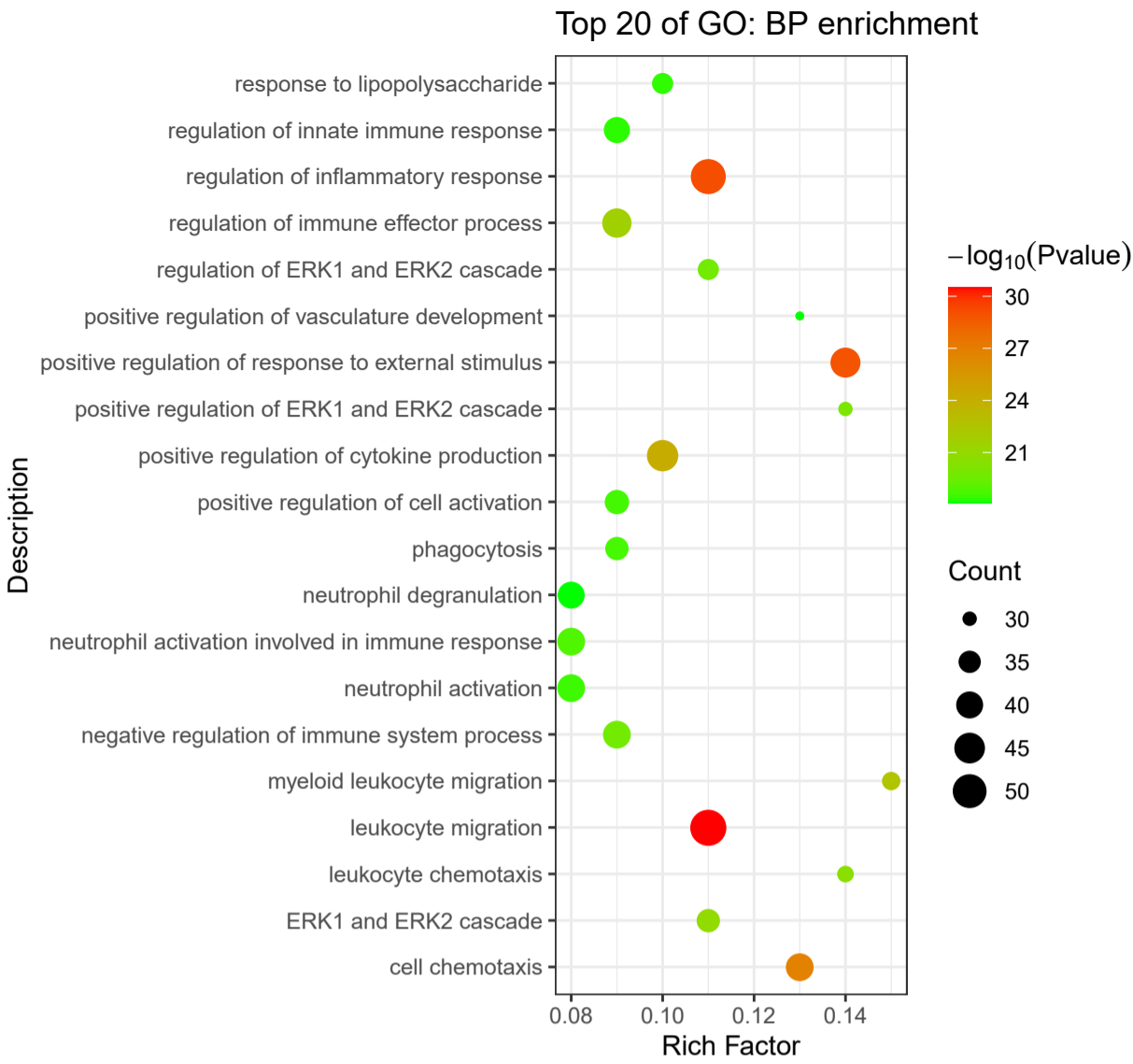

C

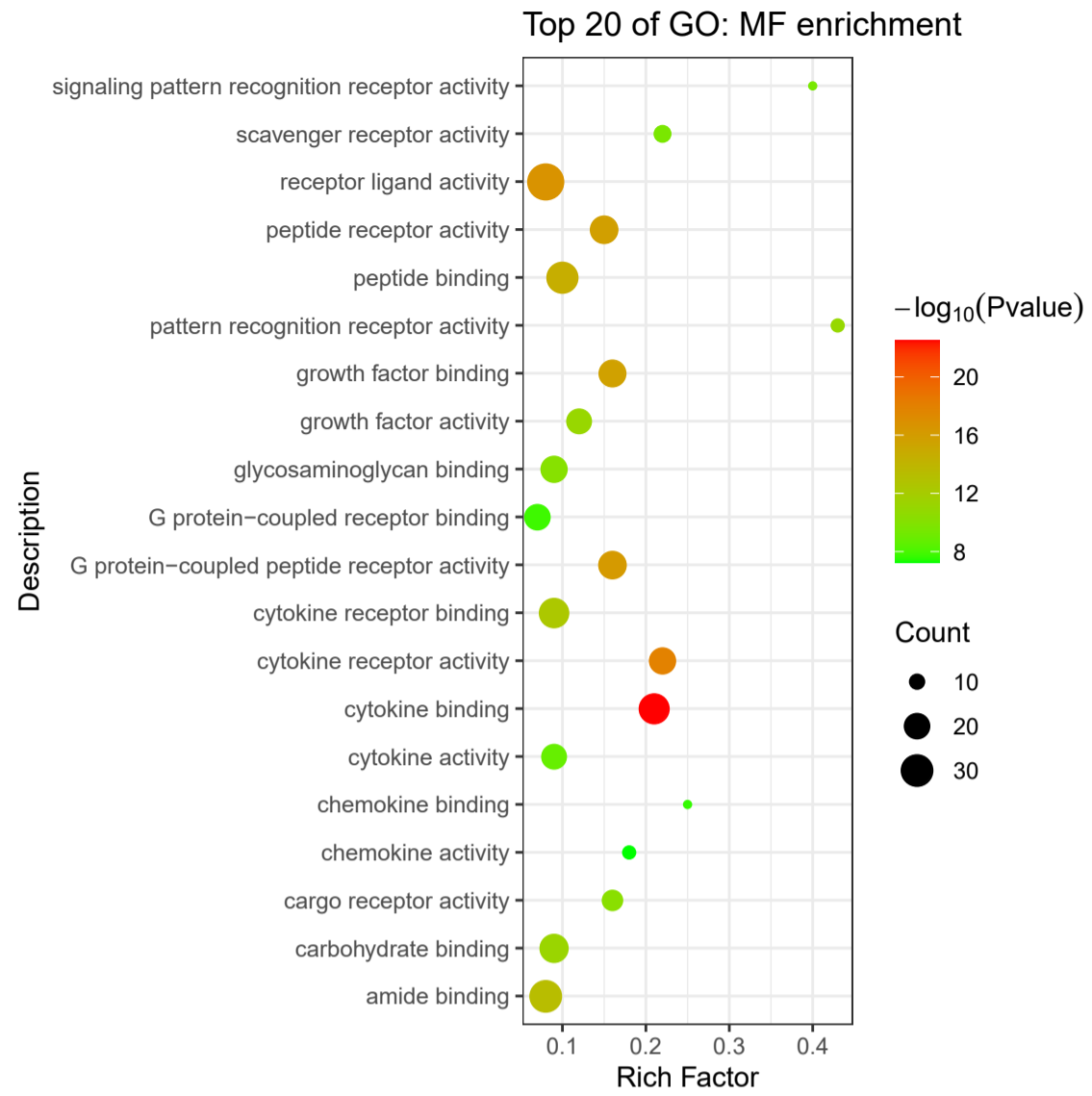

B

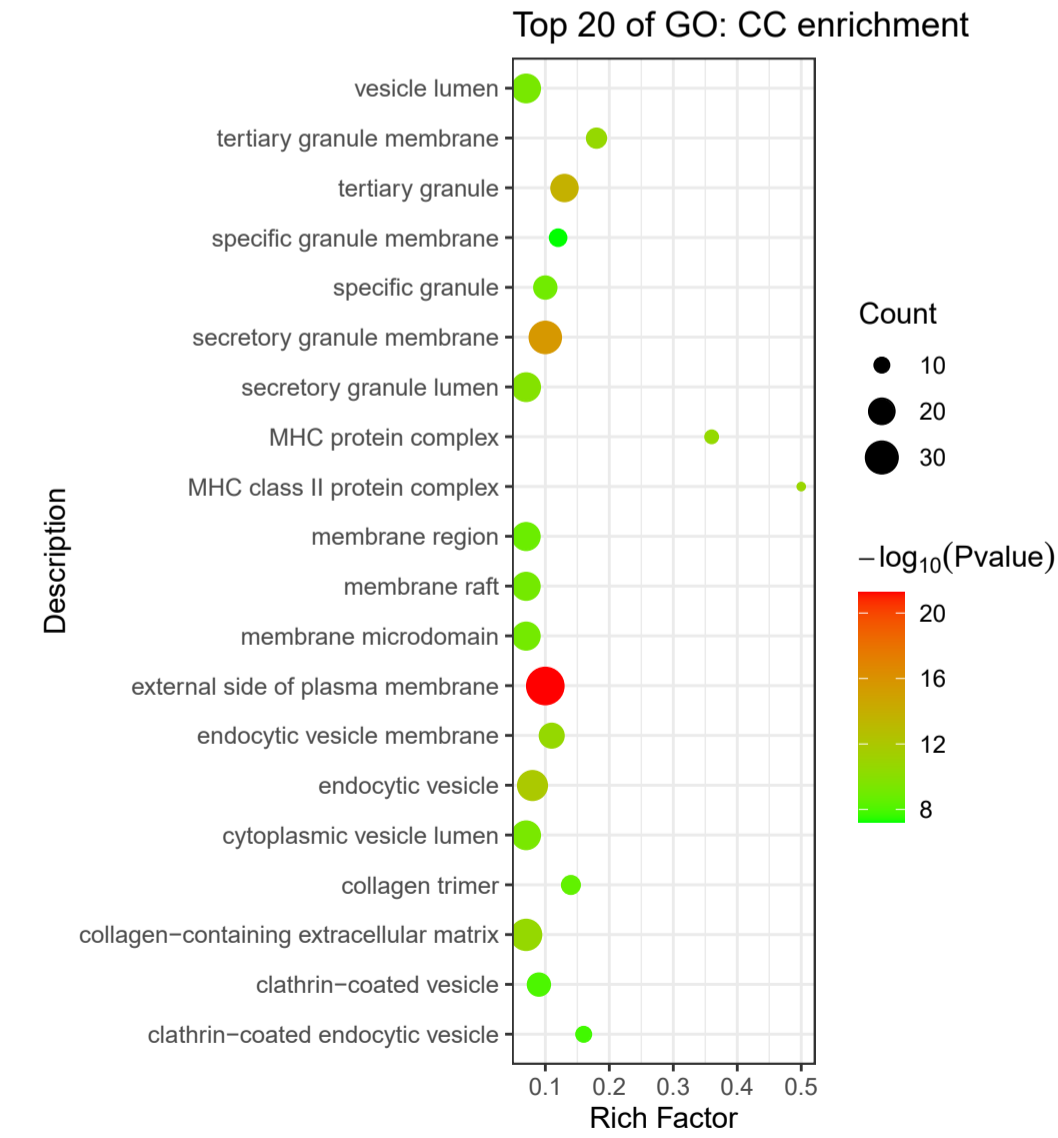

D

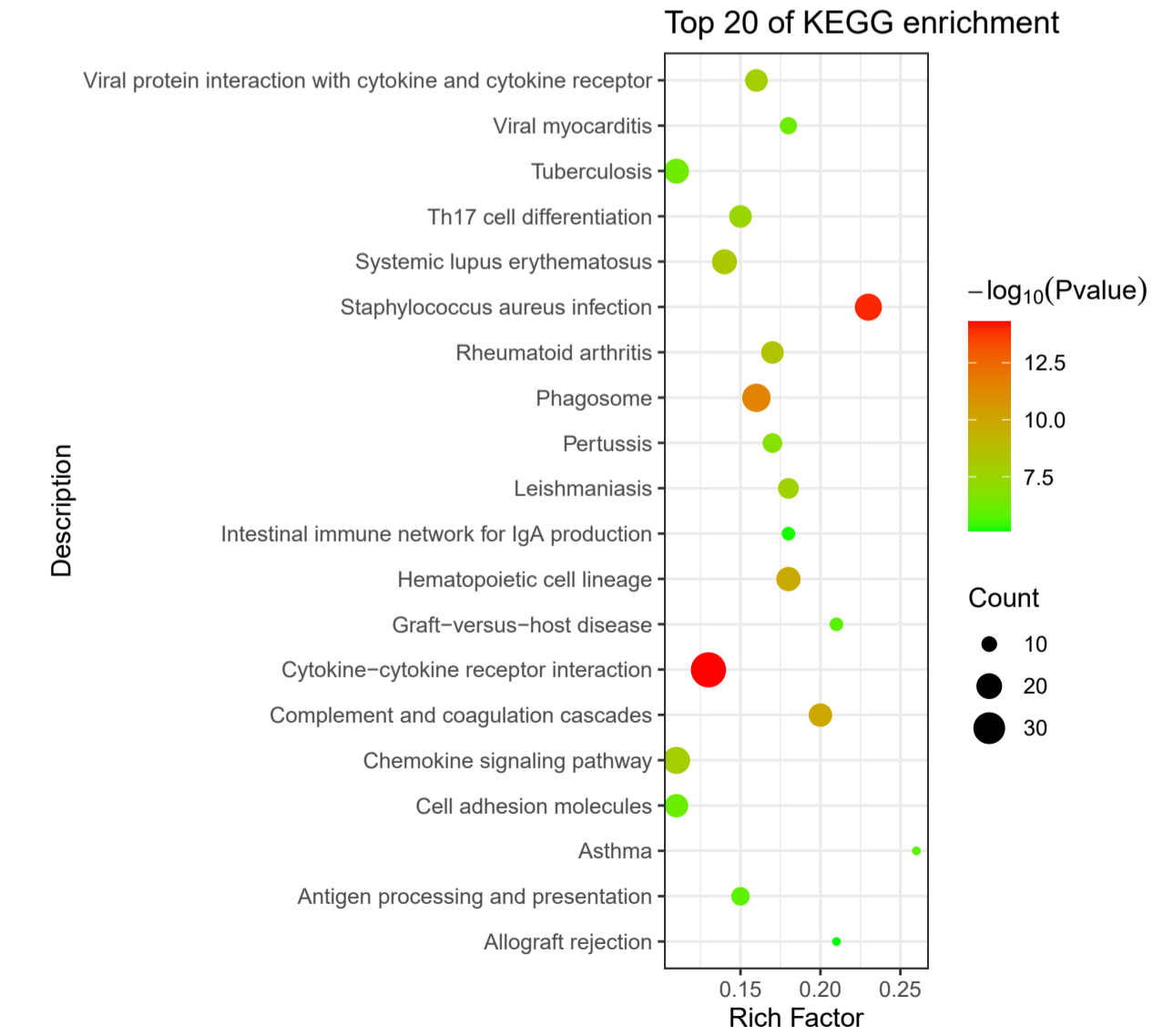

Supplement: Supplementary Figure 1 — Scatter plot of correlation between GS and MM gene in turquoise module. [file DataSheet_1.zip › Supplementary Figures/Supplementary Figure 2.PDF]

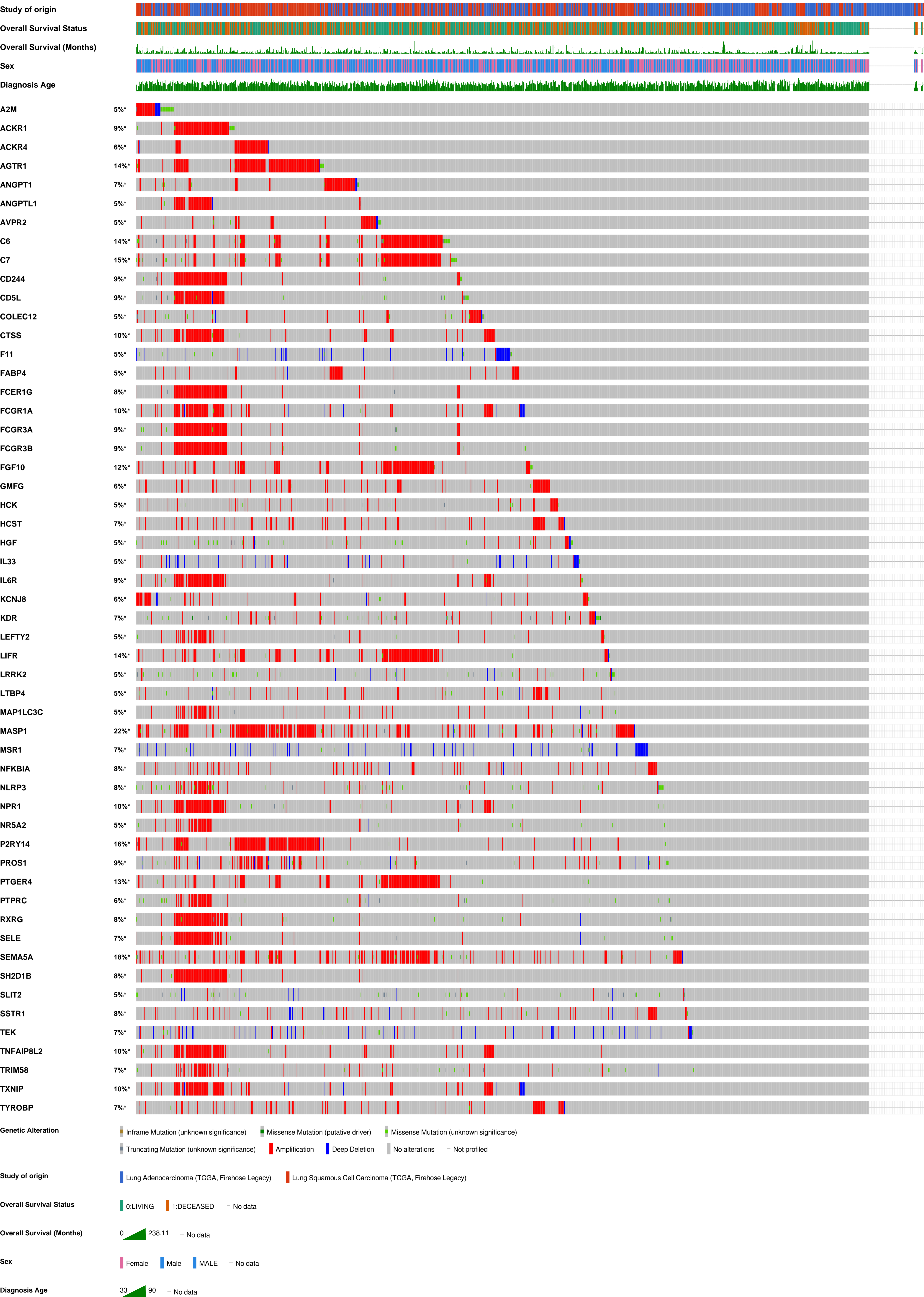

Supplement: Supplementary Figure 1 — Scatter plot of correlation between GS and MM gene in turquoise module. [file DataSheet_1.zip › Supplementary Figures/Supplementary Figure 3.PDF]

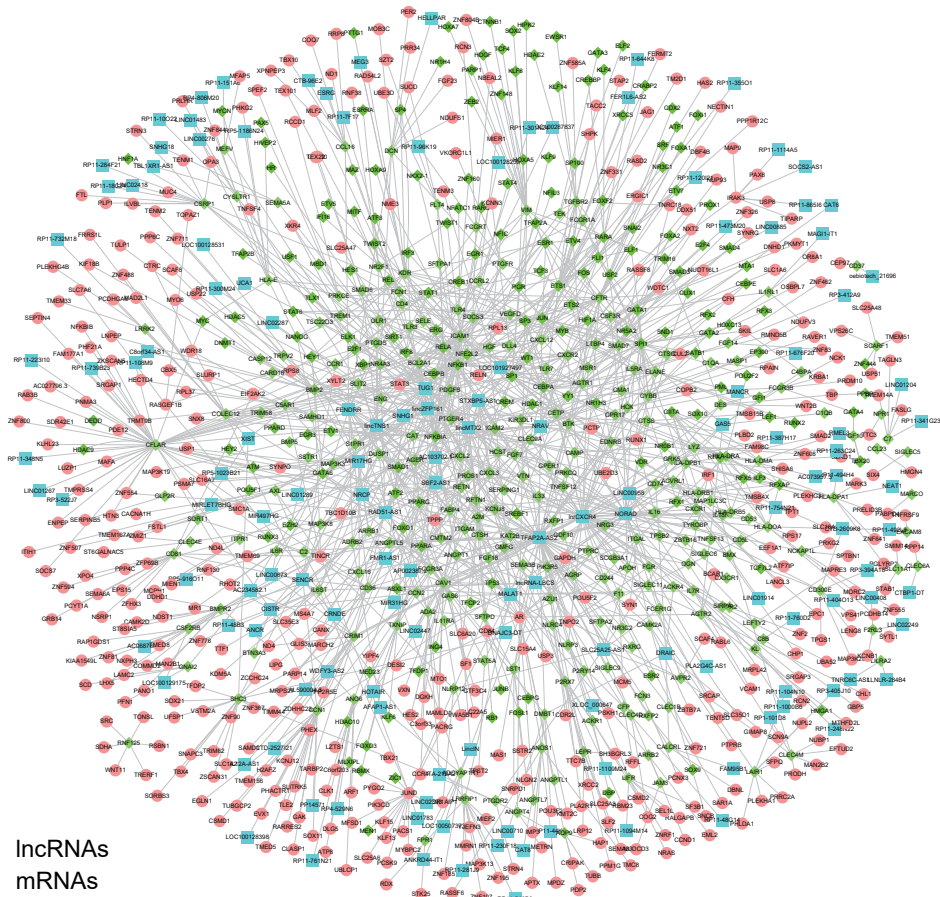

Supplement: Supplementary Figure 1 — Scatter plot of correlation between GS and MM gene in turquoise module. [file DataSheet_1.zip › Supplementary Figures/Supplementary Figure 5.PDF]

A Hazard ratio of 15 genes

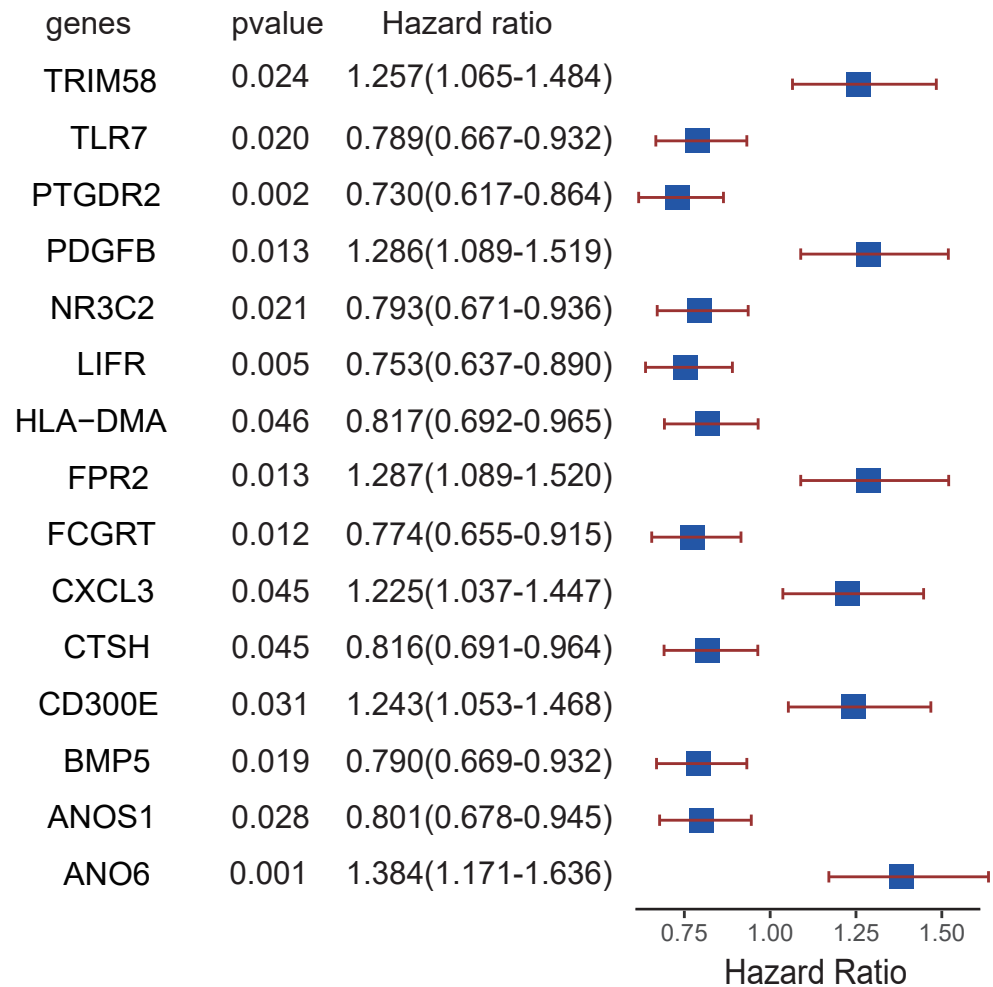

B

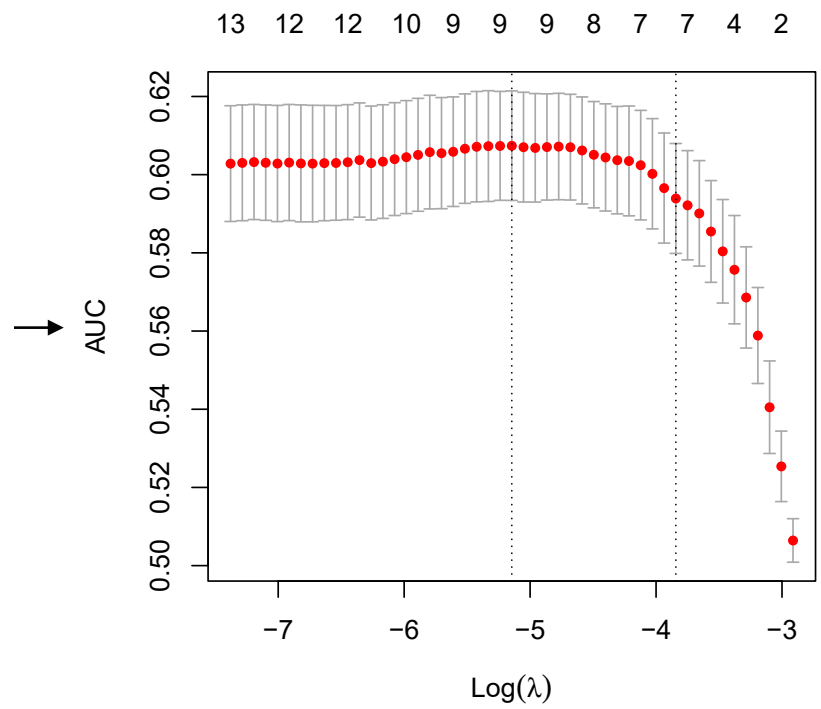

C

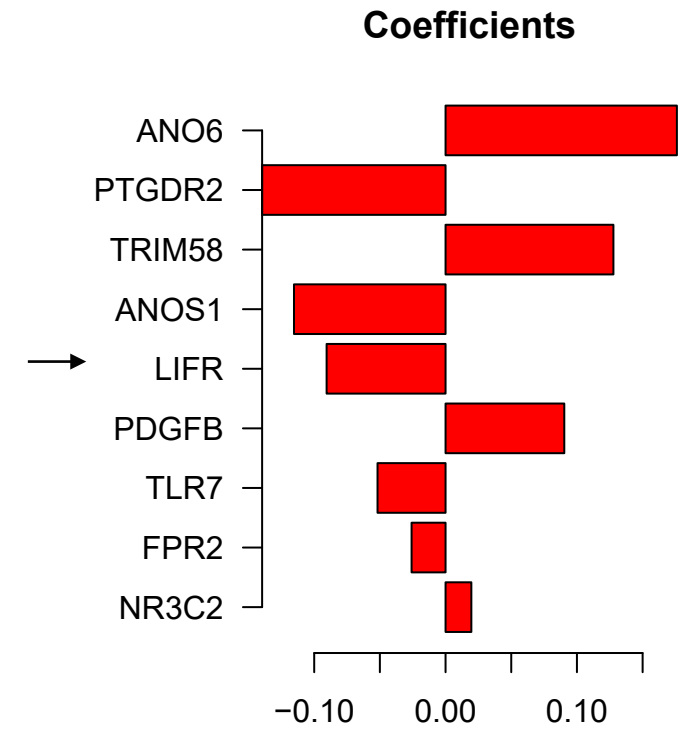

Supplement: Supplementary Figure 1 — Scatter plot of correlation between GS and MM gene in turquoise module. [file DataSheet_1.zip › Supplementary Figures/Supplementary Figure 6.PDF]

A

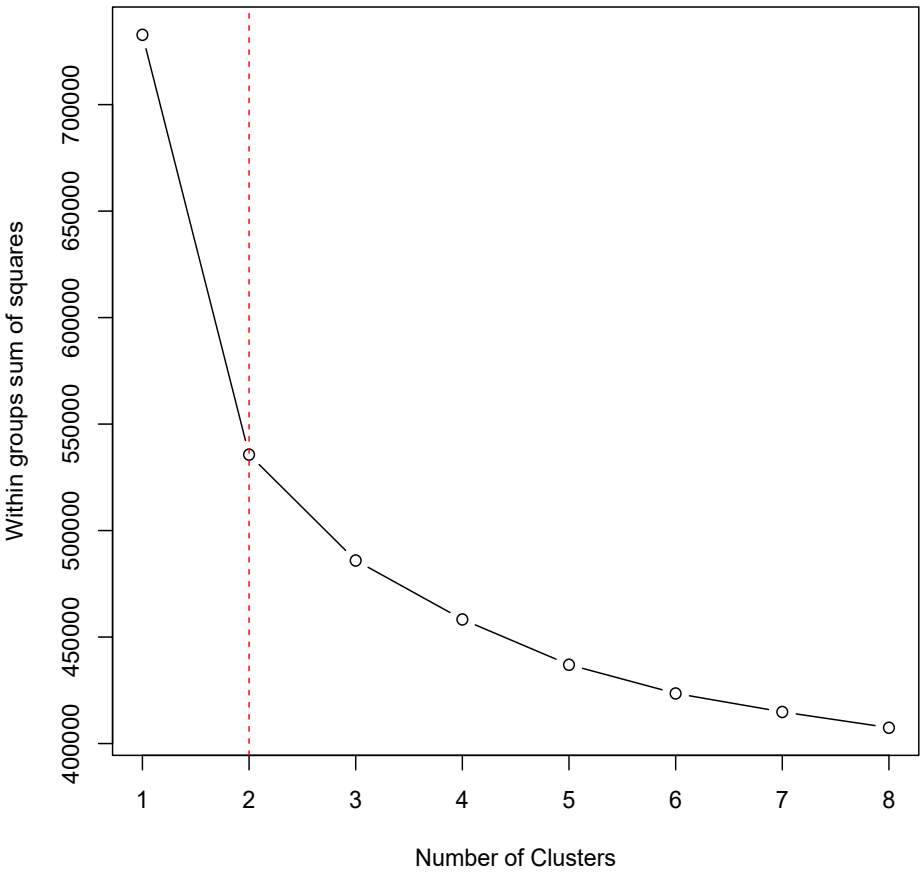

B

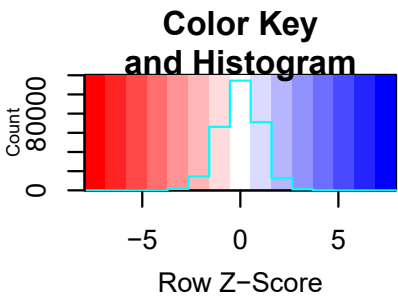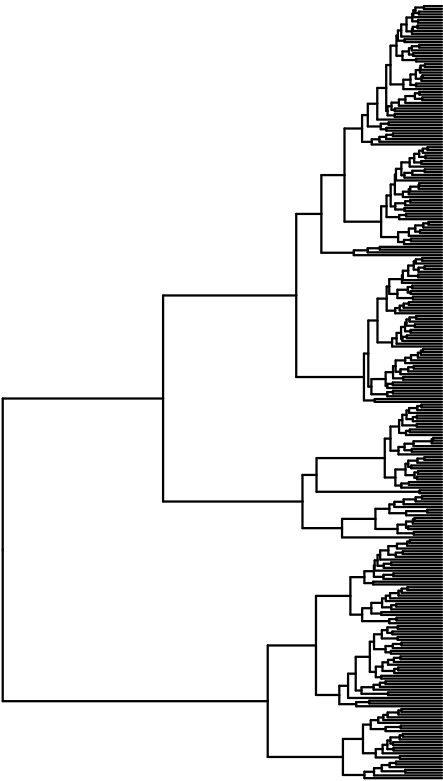

C

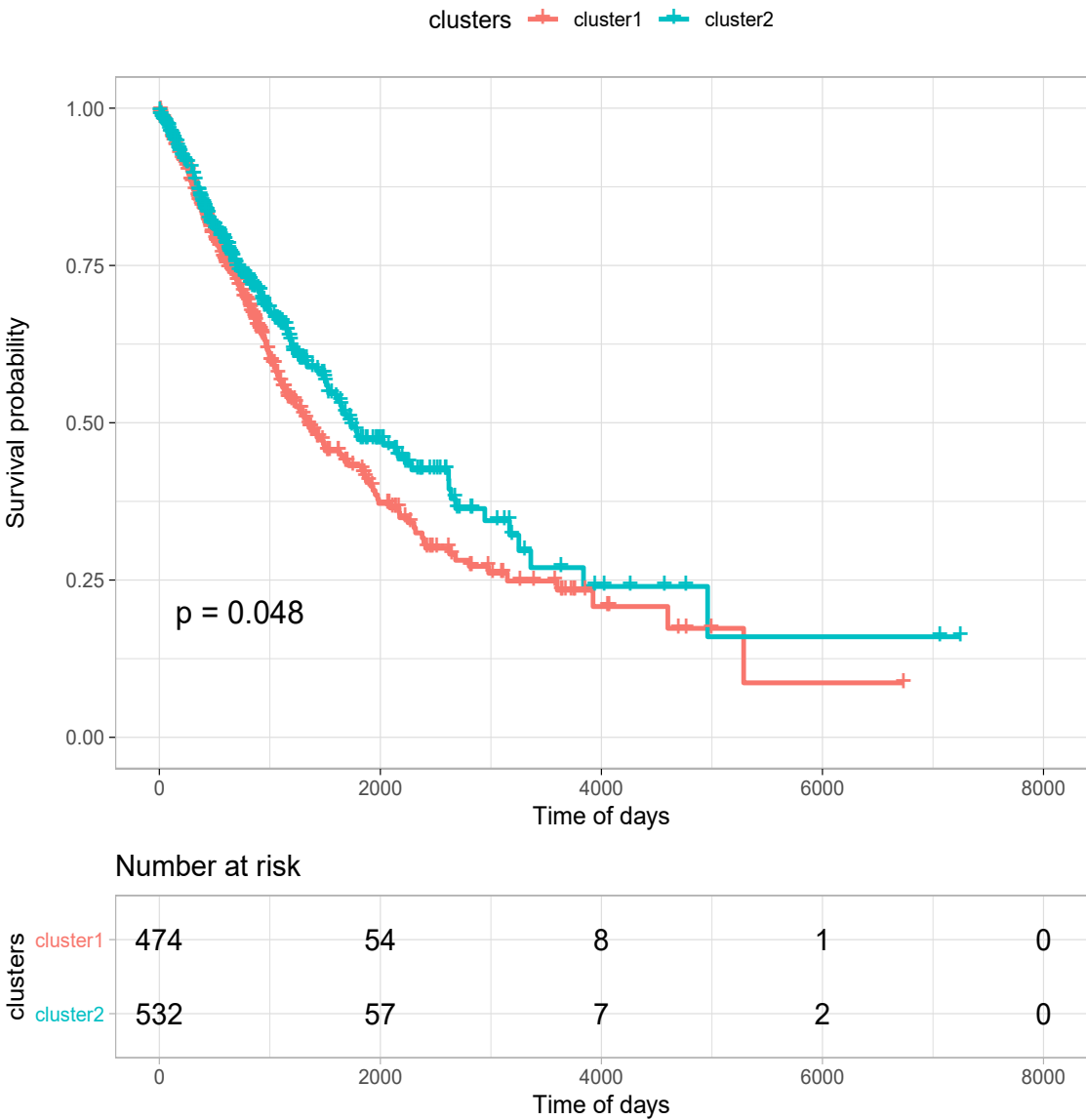

cluster1

cluster2

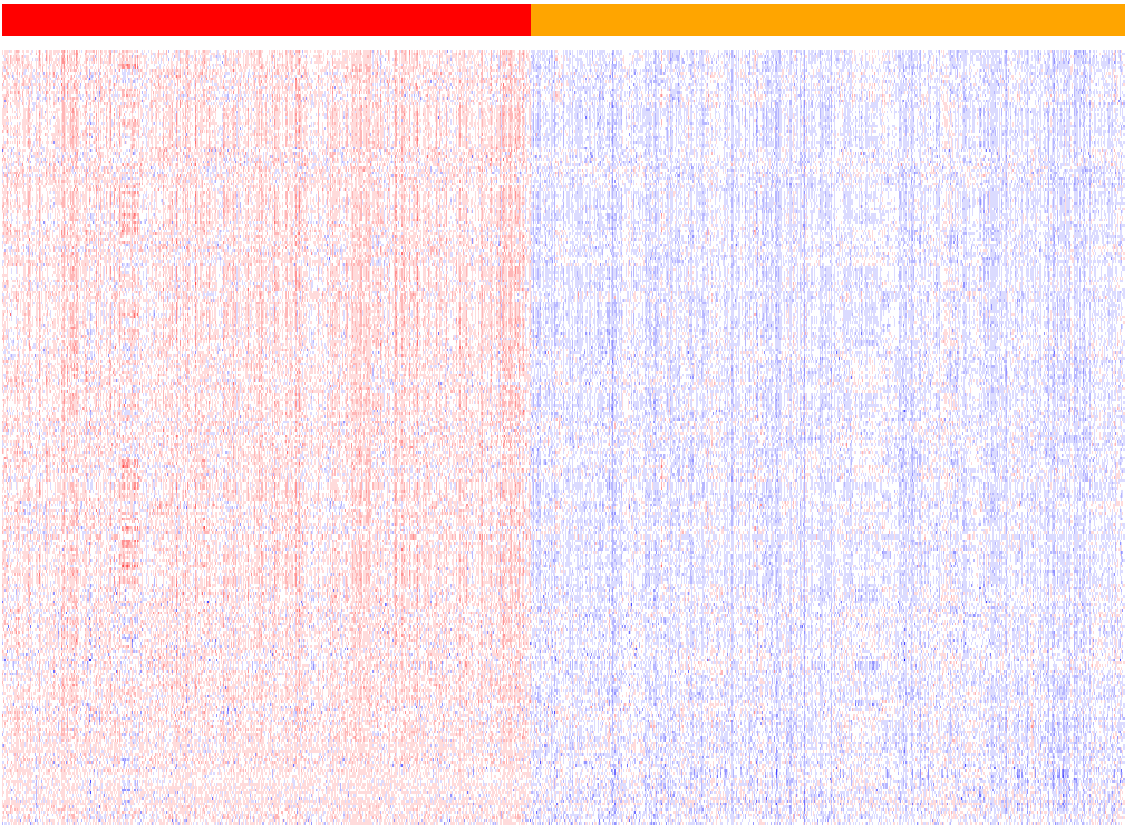

Supplement: Supplementary Figure 1 — Scatter plot of correlation between GS and MM gene in turquoise module. [file DataSheet_1.zip › Supplementary Figures/Supplementary Figure 7.PDF]

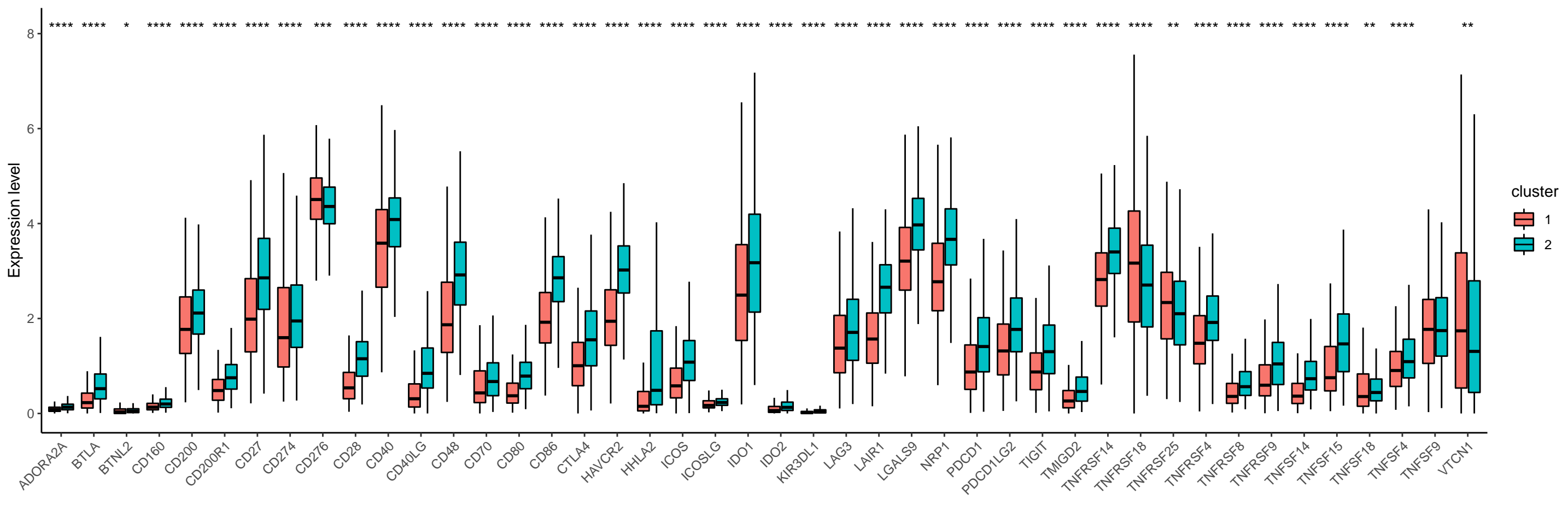

Supplement: Supplementary Figure 1 — Scatter plot of correlation between GS and MM gene in turquoise module. [file DataSheet_1.zip › Supplementary Figures/Supplementary Figure 8.PDF]

cluster 1 2

$1.2e-11$

TIDE

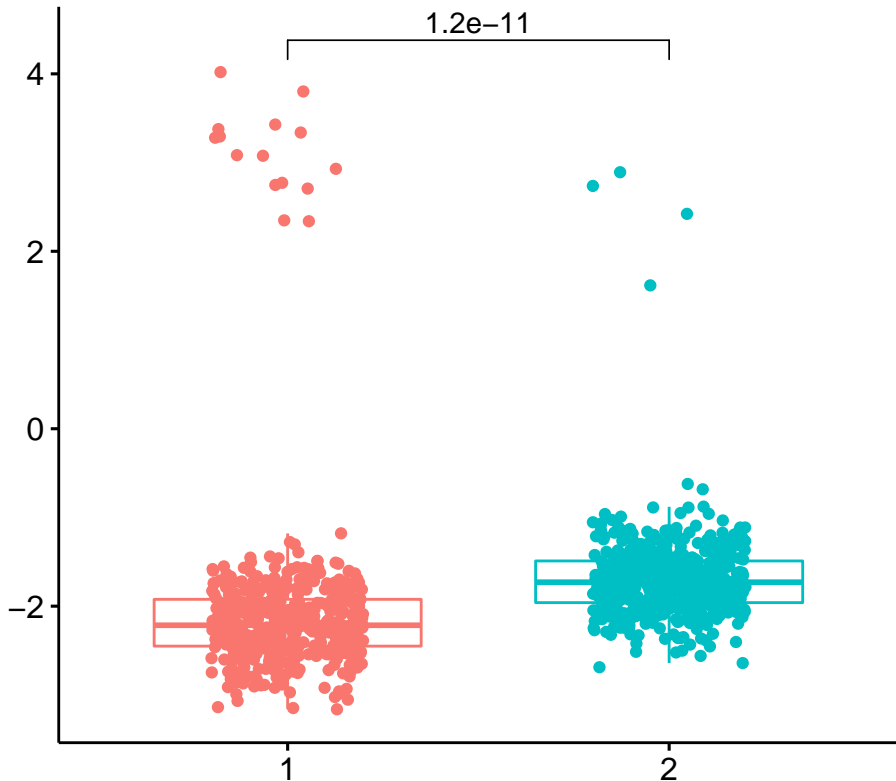

Supplement: Supplementary Figure 1 — Scatter plot of correlation between GS and MM gene in turquoise module. [file DataSheet_1.zip › Supplementary Figures/Supplementary Figure 9.PDF]
