# Supplementary material for: A new immune signature for survival prediction and immune checkpoint molecules in non-small cell lung cancer
Source: Front Oncol. 2023 Jan 30;13:1095313. doi: 10.3389/fonc.2023.1095313 (PMC9924230; doi:10.3389/fonc.2023.1095313)
Supplement: Supplementary Figure 1 — Scatter plot of correlation between GS and MM gene in turquoise module. [file Supplementary Figure 4.PDF › Supplementary Figures/Supplementary Figure 4.PDF]

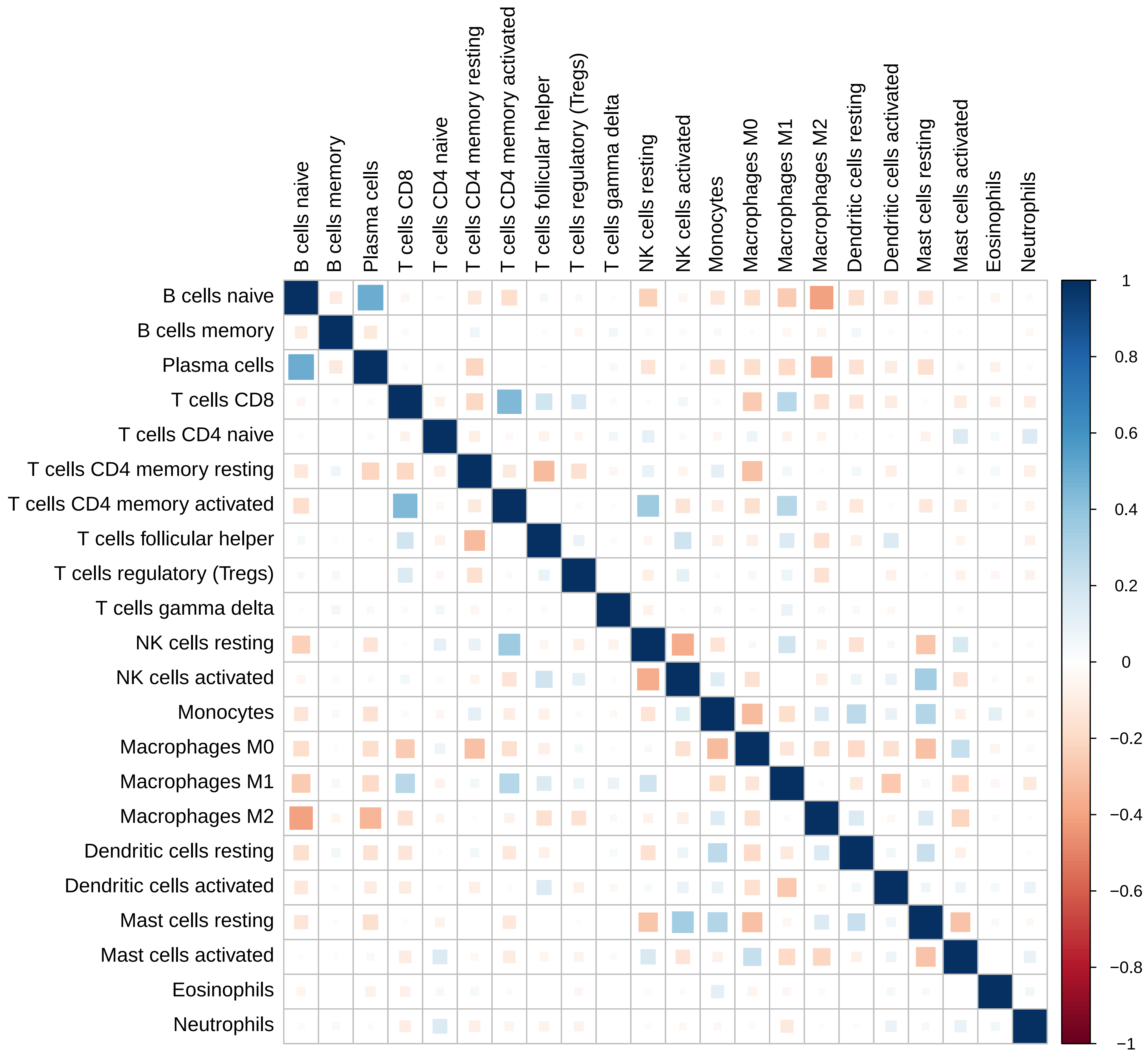

B cells naive
B cells memory
Plasma cells
T cells CD8
T cells CD4 naive
T cells CD4 memory resting
T cells CD4 memory activated
T cells follicular helper
T cells regulatory (Tregs)
T cells gamma delta
NK cells resting
NK cells activated
Monocytes
Macrophages M0
Macrophages M1
Macrophages M2
Dendritic cells resting
Dendritic cells activated
Mast cells resting
Mast cells activated
Eosinophils
Neutrophils
1
0.8
0.6
0.4
0.2
0
−0.2
−0.4
−0.6
−0.8
−1
